# Effect of pelvic floor electrical stimulation on diaphragm excursion and rib cage movement during tidal and forceful breathing and coughing in women with stress urinary incontinence: A randomized controlled trial

**DOI:** 10.1097/MD.0000000000024158

**Published:** 2021-01-08

**Authors:** Ui-jae Hwang, Min-seok Lee, Sung-hoon Jung, Sun-hee Ahn, Oh-yun Kwon

**Affiliations:** a 234 Maeji-ri, Heungeop-Myeon, Wonju, Kangwon-Do, 220–710, Department of Physical Therapy, Graduate School, Yonsei University, Wonju; b Sophie-Marceau Women's Clinic, Daegu; c 234 Maeji-ri, Heungeop-Myeon, Wonju, Kangwon-Do, 220–710, Department of Physical Therapy, College of Health Science, Laboratory of Kinetic Ergocise Based on Movement Analysis, Yonsei University, Wonju, South Korea.

**Keywords:** Breathing, cough, diaphragm, pelvic floor muscle

## Abstract

**Background:**

The pelvic floor muscle (PFM) is associated with respiratory function. We investigated the effects of PFM training by pelvic floor electrical stimulation (PFES) on PFM strength, diaphragm excursion, and upper rib cage movement during tidal and forceful breathing and coughing in women with stress urinary incontinence (SUI).

**Methods:**

In total, 33 participants with SUI were divided into PFES and control groups. The two groups were measured pre- and post-8 weeks of training. Diaphragm excursion and upper rib cage movement during tidal and forceful breathing and coughing and PFM strength were measured using sonography, electromagnetic sensors, and perineometry.

**Results:**

There were significant difference of main effect between pre- and post-training and between groups in PFM strength (between groups: *P* = .001, between time: *P* < .001) and diaphragm excursion during forceful breathing (between groups: *P* = .015, between time: *P* = .026) and coughing (between groups: *P* = .035, between time: *P* = .006). There were significant differences in diaphragm excursion during tidal (*P* = .002) and forceful breathing (*P* = .005) and coughing (*P* < .001) between pre- and post-training in the PFES group. Elevation of the upper rib cage during tidal (*P* < .001) and forceful breathing (*P* = .001) was significantly decreased after 8 weeks of training in the PFES group. Widening in the horizontal plane in the upper rib cage during forceful breathing (*P* < .001) was significantly increased after 8 weeks of training in the PFES group. PFM strength (*P* < .001) was significantly increased after 8 weeks of training in the PFES group.

**Conclusions:**

Pelvic floor muscles training by electrical stimulation can improve diaphragm excursion and breathing patterns in women with SUI.

## 1 Introduction

Stress urinary incontinence (SUI) has been defined by the International Continence Society as “the complaint of involuntary leakage on exertion, coughing or sneezing”.^[1]^ Pelvic floor muscle (PFM) training is the first line treatment in SUI treatment, increasing PFM strength and urethral pressure, and decreasing urethral hypermobility.^[2,3]^ Pelvic floor electrical stimulation (PFES), as a form of PFM training, could improve urinary loss and increase the strength of PFM contractions by identifying the PFM or facilitating the ability to volitionally contract the PFM.^[4–6]^

An abnormal breathing pattern can be activated in patients with SUI and PFM dysfunction;^[7–9]^ during inspiration, the rib cage elevates more and the diaphragm descends less than in healthy individuals.^[7]^ There is often less abdominal wall excursion and more rib cage elevation in patients with SUI.^[7]^ During expiration, the rib cage drops down, the abdominal wall is forced out, and the PFM is forced down.^[7]^

In normal breathing, a costodiaphragmatic breathing pattern is monitored when the lateral costal and abdominal expansion is predominant over the superior thoracic expansion during inspiration at rest, to allow maximum lung expansion and gas exchange.^[7,10,11]^ An abnormal breathing pattern occurs when the superior thoracic expansion exceeds the lateral costal abdominal and lateral costal expansion during inspiration at rest, known as the upper costal breathing type.^[7,10,11]^ Previous studies suggested theoretically that this abnormal breathing type produces a smaller expansion of the rib cage.^[10,11]^ Although the pectoralis major, pectoralis minor, latissimus dorsi, and upper trapezius are considered more accessary respiratory muscle than postural function and contribute to upper costal breathing pattern in the dysfunctional or paradoxical breather.^[12]^ An inhibited diaphragmatic movement could impair the coordination between the diaphragm, transverse abdominis muscle and PFM during tasks or functional movement.^[13–15]^

The PFM is associated with respiratory function.^[16–19]^ It contracts eccentrically during inspiration and contracts concentrically together with the abdominal muscles during forced expiratory maneuvers and coughing, thereby reducing the volume of the abdominal cavity and increasing intra-abdominal pressure (IAP), which forces the diaphragm upwards and increases expiratory effort.^[14,16,18,19]^ The PFM works in synergy with the diaphragm, to control and respond to changes in IAP, provide trunk stability, and contribute to continence while breathing and coughing.^[14,16,18,20,21]^ The core muscle training included PFM contraction affects breathing movements measured by the respiratory inductive plethysmography.^[22]^ Because of the association between the PFM and breathing, as an alternative treatment for urinary incontinence, not only PFM training but also corrective training for breathing patterns and diaphragmatic breathing training have been suggested^[7]^ and applied.^[23]^ However, although previous studies suggested association PFM and breathing,^[16–19]^ causality is still unclear for relationship between PFM and breathing and there are lack of study for effect of PFM training on breathing pattern. Thus, it is necessary to study whether PFM training affects diaphragm contraction and breathing patterns.

We investigated the effects of PFM training by PFES on PFM strength, diaphragm excursion, upper rib cage movement during tidal and forceful breathing, and coughing in women with SUI.

## 2 Methods

### 2.1 Subjects and design

The study design was a single blinded randomization of all participants into PFES and control group in a laboratory setting, from August to December 2018. G∗Power (version 3.1.3; University of Trier, Trier, Germany) was used to calculate the sample size for a power of 0.95, an α level of 0.05, partial η^2^ of 0.387 and an effect size f of 0.794, as determined by pilot data (3 participants each group) on variables of diaphragm excursion during forceful breathing. The sample size required at least 12 subjects per group. Participants were recruited by advertisements that provided a telephone contact; all participants were asked to visit the Urogynecology Clinic in Seoul, Korea, for diagnosis of SUI, and were evaluated regarding the inclusion and exclusion criteria.

**Figure 1:**
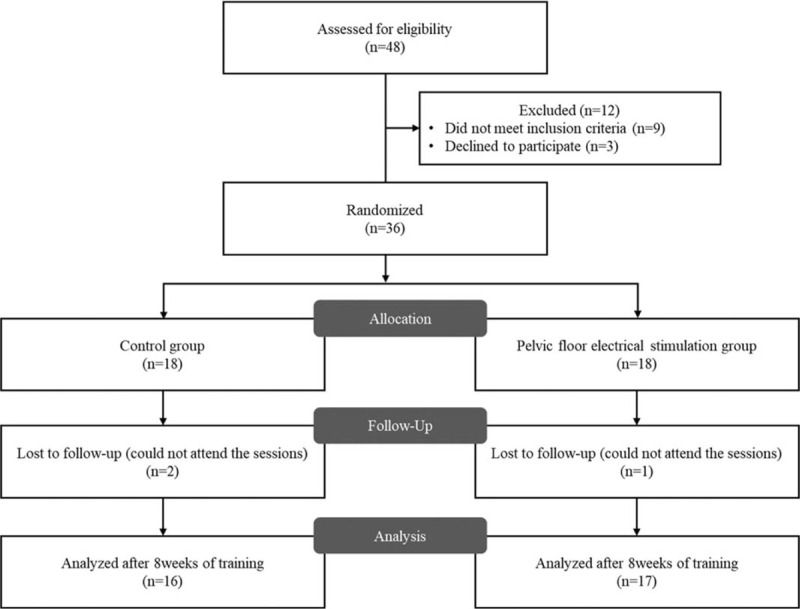
Flow diagram of participant recruitment.

Table 1 shows the inclusion/exclusion criteria. In total, 33 subjects were allocated using a random numbers generated by www.randomization.com into PFES and control group (Fig. 1). All subjects gave written informed consent in a form approved by the Institutional Review Board of Yonsei University Mirae Campus (approval no. 1041849–202002-BM-014-01), before the study. The study protocol was registered with the Clinical Research information Service (KCT0003357).

**Table 1:** Inclusion and exclusion criteria.

Inclusion criteria
Age between 30 and 60 years
Body mass index < 30 kg/m^2^
Leakage episode recorded more than once a week
SUI diagnosed by a urogynecologist
Successful completion of the medical screening questionnaire
Not addicted to alcohol or drugs
Exclusion criteria
Not meeting the inclusion criteria
Concomitant treatment for SUI during the trial period
Cardiac pacemaker or metal materials implanted
Pelvic or abdominal surgery within the last 6 months
Pregnant/planning to become pregnant
Aversion to electrical stimulation
Urinary tract infection
Urogenital prolapse of grade III or higher
Neurological or psychiatric disease
Cognitive impairment: perception problem of understanding of the experimental procedure

### 2.2 Pelvic floor electrical stimulation

The PFES device (EasyK7, Alphamedic Co., Ltd., Daegu, Korea) applied 3 surface electrodes [positioned in both the sacral (1 electrode) and perivaginal regions (2 electrodes)] for stimulating the PFM and surrounding structures. These electrodes create the electromagnetic field that stimulates the PFM when the user sits on the device. For neuromuscular electrical stimulation, high-frequency (50–75 Hz) fatigue generates excessive loss of force, which could be due to failure of electrical propagation with a rapid decreasing in the evoked action potential amplitude.^[24,25]^ Thus, we decided to use electrical stimulation at 25 Hz. The PFES was applied as biphasic and asymmetric impulses at 25 Hz;^[26,27]^ the pulse and resting durations were 11 s each. A PFES session was 15 minutes in duration.

### 2.3 Intervention

The PFES participants were each given a PFES device, and were taught how to use, apply, and manage the device in our laboratory setting. We asked participants to use the PFES device once a day (15-minutes session), 5 days a week for 8 weeks.^[28]^ Also, all subjects underwent training sessions exploring possible increases in PFES amplitude (as individually tolerated). Adherence to this schedule was confirmed by phone twice each week and we encouraged participants to perform PFES at least >5 days each week.

The control group walked for >20 minutes daily. Both groups were assessed at baseline and after 8 weeks.

## 3 Outcome measures

### 3.1 Pelvic floor muscle strength

PFM strength was measured in the hook-lying by a urogynecologist, using a VVP-3000 perineometer (QLMED Ltd, Gyeonggi-do, Korea), and a vaginal probe 115 mm in length (vaginal insertion surface measurement length 66 mm) and 24 mm in diameter.^[28,29]^ The initial pressure of the vaginal probe was controlled at 40 mm Hg, because of differences in the vaginal volume of each subject. The baseline without voluntary PFM contraction was recorded in mmHg and then the device was zeroed during rest. The PFM strength was measured from the baseline to that of peak effort for 5 s and recorded (in mm Hg) as the mean increase in vaginal pressure during 2 maximal voluntary contractions.

### 3.2 Diaphragm excursion by M-mode ultrasonography

Subjects were positioned supine and diaphragm excursions were recorded in the M-Mode. The ultrasound device (A35; Samsung Medison, Seoul, Korea) was used to measure ultrasonographic indices of the diaphragm with a sector transducer (3.5 MHz). The probe was placed on the abdominal region just below the lowest right rib, between the mammillary and midaxillary lines in the longitudinal plane in the superior direction, with the liver as an acoustic window.^[30]^ The angle of the probe was adjusted so that the ultrasound beam was perpendicular to the posterior third of the right hemidiaphragm.^[30]^ The diaphragm excursion was assessed from the M-mode image between the end of expiration and end of inspiration during tidal and forceful breathing and coughing and reported in cm. The averages of 3 values were taken for each tidal and forceful breath and coughing.

### 3.3 Kinematics measurement for upper rib cage

**Figure 2:**
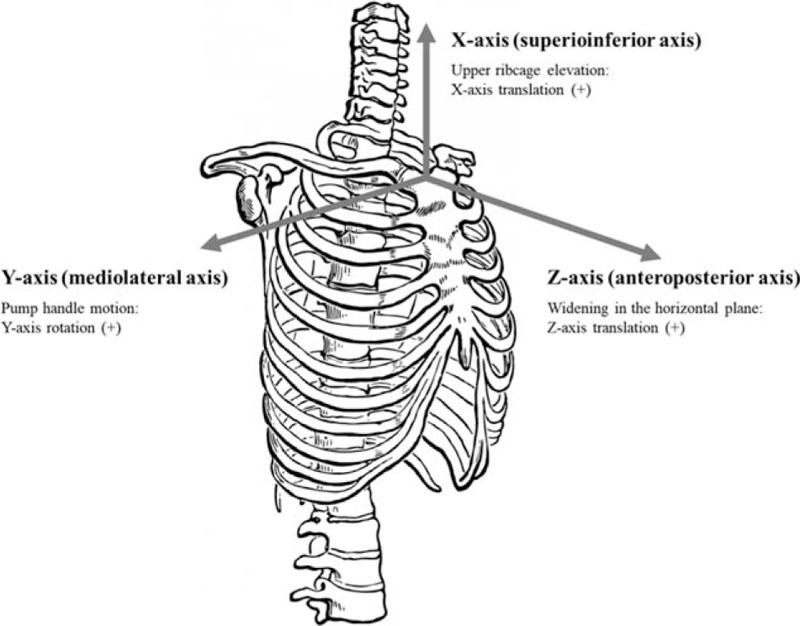
Axes of upper rib cage movement.

The Liberty system (Polhemus, VT, USA) was used to investigate upper rib cage movement at 120 Hz and to monitor superoinferior and anteroposterior translation and pump-handle movement (anteroposterior rotation) during tidal and forceful breathing and coughing. The electromagnetic motion sensor was attached at the center of the manubrium below the jugular notch. Metal objects were removed to avoid interference. The sensor and wire were firmly fixed with adhesive tape in the same position to prevent motion artifacts. The transmitter was placed in the same position and orientation during tidal and forceful breathing and coughing. The electromagnetic tracker system was aligned with the orientation of breathing in the supine position, with +x directed along the superoinferior axis, +y directed along the mediolateral axis, and +z directed along the anteroposterior axis (Fig. 2). The sensor in the center of the manubrium was used for superoinferior and anteroposterior translation (cm) and anteroposterior rotation data (°) of the upper rib cage. Upper rib cage movements (translation and rotation) were measured based on the difference between the end of expiration and end of inspiration during tidal and forceful breathing and coughing. The averages of 3 values were taken for each tidal and forceful breath and coughing.

### 3.4 Statistical analyses

The Kolmogorov–Smirnov Z-test was applied to verify the assumption of data normality. Initially, an independent *t*-test was performed for each dependent variable (PFM strength, diaphragm excursion, upper rib cage movement during tidal and forceful breathing and coughing) to determine whether there were differences pre-measurement between the groups. When statistically significant differences were found, analysis of covariance (ANCOVA) was performed with the baseline measurement as a covariate. When no statistically significant differences were found, two-way analysis of variance (ANOVA) with repeated measures was conducted to compare measurements of each dependent variable between groups, as well as the interaction effect (time × group). Whenever a significant interaction was observed, a paired t-test was used to determine within-group differences, and an independent t-test was used to determine between-group differences. All statistical analyses were performed using SPSS ver. 18.0 software (SPSS Inc., Chicago, IL, USA). A *P* value of .05 was taken to indicate statistical significance.

## 4 Results

The characteristics of the subjects are shown in Table 2. There were no adverse events during performing the present study. The mean intensity among all participants was 19.13 ± 5.47 mA (range: 2.5 to 30 mA). Compliance to the intervention was to perform PFES for 5 days a week. Three participants (2 participants in control group and 1 participant in PFES group) did not perform compliance to the intervention (Fig. 1). Table 3 shows the post-intervention changes in PFM strength, diaphragm excursion, X- and Z-axis translation, and Y-axis rotation in upper rib cage kinematics during tidal and forceful breathing and coughing relative to baseline for each group.

**Table 2:** Characteristics of the participants.

	Control group (n = 16)	PFES group (n = 17)	*P* value
Age (y)	41.1 ± 7.2	42.1 ± 8.8	.726
BMI (kg/m^2^)	22.8 ± 3.5	22.6 ± 2.7	.869
Duration of symptoms (y)	7.8 ± 6.0	5.9 ± 3.6	.229
Deliveries (n)	1.5 ± 0.9	1.8 ± 0.8	.385
Vaginal deliveries (n)	1.5 ± 0.9	1.4 ± 1.0	.799

**Table 3:** PFM strength, diaphragm excursion and upper rib cage kinematics values pre- and post-training for both groups (means ± SD).

		Group	Pre	Post	*P*
Pelvic floor muscle strength		PFES	24.21 ± 7.14	39.14 ± 11.88	.000^∗^
		Control	33.19 ± 13.18	29.31 ± 11.89	.072
Diaphragm excursion (cm)	Tidal breathing	PFES	1.79 ± 0.81	2.31 ± 0.83	.002^∗^
		Control	1.82 ± 0.79	1.70 ± 0.73	.393
	Forceful breathing	PFES	6.26 ± 0.95	6.73 ± 1.05	.005^∗^
		Control	5.68 ± 0.98	5.65 ± 0.91	.829
	Coughing	PFES	4.25 ± 1.01	5.04 ± 0.91	.000^∗^
		Control	4.05 ± 0.98	3.83 ± 0.94	.057
X-axis translation in upper rib cage kinematics (cm)	Tidal breathing	PFES	0.09 ± 0.03	0.06 ± 0.02	.000^∗^
		Control	0.14 ± 0.17	0.18 ± 0.19	.204
	Forceful breathing	PFES	1.02 ± 0.58	0.77 ± 0.44	.001^∗^
		Control	1.07 ± 0.42	1.16 ± 0.38	.358
	Coughing	PFES	2.25 ± 0.99	2.03 ± 0.78	.400
		Control	2.38 ± 0.77	2.27 ± 0.85	.610
Z-axis translation in upper rib cage kinematics (cm)	Tidal breathing	PFES	0.09 ± 0.05	0.16 ± 0.23	.218
		Control	0.15 ± 0.20	0.11 ± 0.06	.513
	Forceful breathing	PFES	0.81 ± 0.35	1.03 ± 0.35	.000^∗^
		Control	0.93 ± 0.20	0.86 ± 0.38	.075
	Coughing	PFES	1.08 ± 0.41	0.97 ± 0.45	.337
		Control	1.16 ± 0.41	1.18 ± 0.37	.866
Y-axis rotation in upper rib cage kinematics (°)	Tidal breathing	PFES	0.73 ± 0.46	0.78 ± 0.43	.751
		Control	0.87 ± 0.64	0.86 ± 0.86	.983
	Forceful breathing	PFES	4.92 ± 3.48	4.99 ± 3.58	.932
		Control	4.75 ± 2.23	4.69 ± 2.67	.936
	Coughing	PFES	11.52 ± 4.24	12.42 ± 4.75	.461
		Control	10.67 ± 3.37	9.48 ± 4.77	.054

### 4.1 Pelvic floor muscle strength

PFM strength was significantly different between groups at baseline. The baseline measurement was used as a covariate in the subsequent analyses. ANCOVA showed a statistically significant main effect between pre- and post-training (95% CI: 2.394–8.726, *P* < .001) and between groups (95% CI: 4.267–12.508, *P* = .001). There were also statistically significant interactions between time and groups (*P* < .001) but no difference between time and the covariate (*P* = .134). The PFES group showed significantly greater PFM strength than the control group (95% CI: −18.277.267–−1.388, *P* = .024) post-training. PFM strength (95% CI: −20.070–−9.794, *P* < .001) was significantly increased after training in the PFES group, while the control group showed no significant difference between pre- and post-training (CI: −0.398–8.156, *P* = .072).

### 4.2 Diaphragm excursion

There were no significant differences between groups at baseline for diaphragm excursion during tidal and forceful breathing and coughing. ANOVA showed a statistically significant main effect between pre- and post-training during tidal (95% CI: 0.007–0.394, *P* = .043) and forceful breathing (95% CI: 0.028–0.414, *P* = .026) and coughing (95% CI: 0.088–0.479, *P* = .006) and between groups during forceful breathing (95% CI: 0.172–1.500, *P* = .015) and coughing (95% CI: 0.053–1.361, *P* = .035). However, there were significant time × group interactions for diaphragm excursion during each action. The PFES group had significantly greater excursion during each action than the control group post-training. There were also significant differences in each action between pre- and post-training, while the control group did not show any significant difference between pre- and post-training.

### 4.3 Upper rib cage kinematics

There were no significant differences between groups at baseline for X- and Z-axis translation, and Y-axis rotation in the upper rib cage during tidal and forceful breathing and coughing. For X-axis translation, ANOVA did not showed a statistically significant main effect between pre- and post-training during tidal and forceful breathing and coughing and between groups forceful breathing and coughing. However, there was significantly difference between groups during tidal breathing (95% CI: 0.001–0.172, *P* = .048). However, there were significant time × group interactions for X-axis translation during tidal (*P* = .022) and forceful breathing (*P* = .005). The PFES group had significantly less X-axis translation during tidal (95% CI: 0.026–0.217, *P* = .014) and forceful breathing (95% CI: 0.096–0.680, *P* = .011) than the control group at post-training. X-axis translation during tidal (95% CI: 0.019–0.043, *P* < .001) and forceful breathing (95% CI: 0.111–0.384, *P* = .001) significantly decreased after 8 weeks of training, while the control group showed no significant differences between pre- and post-training.

For Z-axis translation, ANOVA did not showed a statistically significant main effect between pre- and post-training and between groups during tidal and forceful breathing and coughing. There were significant time × group interactions for Z-axis translation in the upper rib cage during forceful breathing (*P* < .001), but no significant difference during tidal breathing (*P* = .177) or coughing (*P* = .461). The PFES group had significantly greater translation than the control group during forceful breathing (95% CI: −0.499–−0.041, *P* = .022) at post-training. This significantly increased after 8 weeks of training, while the control group showed no significant differences between pre- and post-training.

For Y-axis rotation, ANOVA did not showed a statistically significant main effect between pre- and post-training and between groups during tidal and forceful breathing and coughing. There were no significant time × group interactions for Y-axis rotation during tidal and forceful breathing and coughing. No significant differences were found for main effect in Y-axis rotation in the upper rib cage during tidal and forceful breathing and coughing.

## 5 Discussion

PFM training by PFES significantly increased PFM strength and diaphragm excursion during tidal and forceful breathing and coughing in women with SUI. In addition, there were significant differences in X-axis translation in the upper rib cage during tidal and forceful breathing and Z-axis translation at this location during forceful breathing between pre-and post-PFM training by PFES. Although the cause and effect relationship between altered breathing pattern and PFM weakness is controversial, PFM training by PFES could affect diaphragm excursion and breathing patterns in women with SUI. Also, although we did not measure expiratory function, such as forced vital capacity and forced expiratory flows, and gas change, increasing diaphragm excursion and decreasing elevation of upper ribcage movement may help improve to optimal breathing pattern in women with SUI.

A previous study reported diaphragm excursion (tidal breathing: 18.4 ± 7.6 and forceful breathing: 78.8 ± 13.3 mm) in healthy subjects, assessed via sonography.^[30]^ The diaphragm excursions of subjects with SUI in our study (tidal breathing: 18.1 ± 7.7 and forceful breathing: 59.7 ± 9.7 mm) were smaller than those in that previous study, although it is difficult to compare our data directly to those of the previous study.^[30]^ Movement of the diaphragm and the PFM are synchronous cranio-caudal movements.^[18]^ Similar basic movement patterns of the diaphragm and PFM occur during coughing and breathing.^[18]^ PFM activity increases during inspiration, because of the postulated synergistic coactivation of PFM and anterolateral abdominal muscles.^[14]^ In one study, an incremental positive correlation was found between voluntary PFM contraction strength and forced expiratory flow at 25% (*r* = 0.320), 50% (*r* = 0.388), and 75% (*r* = 0.432) of the forced vital capacity in healthy nulliparous women.^[16]^ Sapsford^[7]^ applied a treatment approach to urinary incontinence using diaphragmatic breathing and motor re-learning of functional expiratory patterns. In addition, retraining diaphragmatic, deep abdominal, and PFM-coordinated function involving diaphragm breathing and functional expiratory pattern training improves symptoms of urinary incontinence as an alternative intervention.^[22,23]^ Otherwise, specific motor learning intervention involving PFM contraction for subjects with sacroiliac joint pain can positively change diaphragm kinematics and patterns of respiration.^[9]^

The cause and effect relationship between improved PFM contraction and improved diaphragm excursion has been controversial. However, our study demonstrated that improved PFM strength by PFES increased diaphragmatic excursion during tidal and forceful breathing and coughing, which may be explained as follows. First, it may increase to work synergistically with the PFM and diaphragm.^[16,18,19]^ Because of a synchronous parallel movement of the diaphragm and PFM during tidal and forceful breathing as well as during coughing,^[18,21]^ PFM training could help the diaphragm co-contract with the PFM. Second, improved PFM strength could withstand the greater IAP during inspiration than before PFM training. The PFM contracts eccentrically during inspiration and contracts concentrically together with the abdominal muscles during coughing, sneezing and forceful expiration, thereby decreasing the abdominal cavity volume and increasing the IAP, which forces the diaphragm upwards and enhances expiratory effort.^[13,14,31]^ Thus, diaphragmatic excursion could be increased by improved PFM strength, because the PFM could maintain a higher urethral than vesical pressure with the urethral sphincter during tidal and forceful breathing and coughing. Third, reduction of the fear or stress of urine leakage could increase diaphragmatic breathing. Women with SUI have expressed their concern about body odor from leaking urine.^[32]^ Emotional states such as stress or fear could cause shallow and rapid breathing patterns.^[33]^ PFES has demonstrated improvement of both subjective and objective symptoms in women with SUI.^[28]^ Thus, subjects may experience more diaphragmatic breathing than before training.

The abnormal breathing pattern of lifting the sternum vertically during inspiration, instead of widening the thorax in the horizontal plane and pump handle motion, occurs due to bilateral overactivity in the scalene, trapezius, and levator scapulae musculature.^[10]^ For the abnormal breathing pattern, excessive use of accessory respiratory muscles may be required to compensate for insufficient gas exchange, which could lead to chronic cervical and shoulder overstrain, decreased rib movement, decreased intercostal muscle activation, and an inhibited diaphragm movement.^[10]^ Our study measured superoinferior (X-axis) and anteroposterior (Z-axis) translation (cm) and anteroposterior (Y-axis) rotation data (°) of the upper rib cage for detecting elevation of the upper rib cage, widening in the horizontal plane, and pump handle motion, respectively. Elevation of the upper rib cage was significantly decreased during tidal and forceful breathing, and widening in the horizontal plane was significantly increased during forceful breathing after PFM training. If recruitment of the abdominal muscles and consequently the PFM during breathing is decreased, the inspiratory effort requires upper rib cage elevation.^[7]^ In addition, an improved diaphragm excursion could lead to improvement of the abnormal breathing pattern. This is possibly the reason why PFM training may help improve to optimal breathing pattern.

### 5.1 Methodological limitations

The principal limitations of this study were the lack of electromyography data to assess changes in PFM and accessory respiratory muscle activation during tidal and forceful breathing and coughing. In addition, we did not measure the vital capacity and expiratory flow rates during tidal and forceful breathing. Further study is needed to determine if PFES training can affect the vital capacity and expiratory flow rates during tidal and forceful breathing in women with SUI. Further studies are also needed to determine the change in respiratory-related cranio-caudal movement of the diaphragm and PFM using real-time dynamic magnetic resonance imaging before and after PFM training by PFES. In addition, although we included women of a wide range of ages with SUI, and both pre- and postmenopausal women, studies with larger sample sizes are required.

## 6 Conclusions

Despite these limitations, pelvic floor muscles training by electrical stimulation can improve diaphragmatic excursion during tidal and forceful breathing and coughing in women with stress urinary incontinence, and the upper rib cage movement pattern during tidal and forceful breathing. The results of this study may be useful for developing guidelines for improving diaphragm excursion and decreasing elevation of upper ribcage movement in patients with stress urinary incontinence. Thus, pelvic floor muscles training could be recommended for improving abnormal breathing pattern in women with stress urinary incontinence.

## Acknowledgments

We wish to thank all of the subjects for their time and commitment to the study.

## Author contributions

**Conceptualization:** Ui-jae Hwang.

**Formal analysis:** Min-seok Lee, Sung-hoon Jung, Sun-hee Ahn.

**Funding acquisition:** Oh-yun Kwon.

**Investigation:** Ui-jae Hwang, Min-seok Lee, Sung-hoon Jung, Sun-hee Ahn.

**Methodology:** Min-seok Lee, Sung-hoon Jung, Sun-hee Ahn.

**Project administration:** Oh-yun Kwon.

**Supervision:** Oh-yun Kwon.

**Validation:** Sung-hoon Jung, Sun-hee Ahn.

**Visualization:** Sun-hee Ahn.

**Writing – original draft:** Ui-jae Hwang, Oh-yun Kwon.
